# Bis(μ-hydroxido-κ^2^ *O*:*O*)bis[bis(5-car­boxy­pyridine-2-carboxyl­ato-κ^2^ *N*,*O* ^2^)iron(III)] dihydrate

**DOI:** 10.1107/S1600536813029449

**Published:** 2013-10-31

**Authors:** Wenhai Cao

**Affiliations:** a Qinghai HuangHe Hydropower Development Co. Ltd, New Energy Branch, Xining, 810008, People’s Republic of China

## Abstract

The complete binuclear complex in [Fe_2_(C_7_H_4_NO_4_)_4_(OH)_2_]·2H_2_O, is generated by the application twofold symmetry. The Fe^III^ atom is coordinated by the O atoms of two bridging hydroxyl groups and by two N and two O atoms from two pyridine-2,5-di­carboxyl­ato ligands, forming a distorted octa­hedral geometry. The Fe⋯Fe separation within the dinuclear complex is 3.0657 (4) Å. In the crystal, O—H⋯O and C—H⋯O hydrogen-bonding inter­actions connect the mol­ecules into a three-dimensional supra­molecular network.

## Related literature

For background to the coordination modes of the pyridine-2,5-di­carboxyl­ate ligand, see: Zhang *et al.* (2005 ▶, 2006 ▶); Liang *et al.* (2000 ▶); Wibowo *et al.* (2011 ▶). For iron complexes of the pyridine-2,5-di­carboxyl­ate ligand, see: Shi *et al.* (2011 ▶); Xu *et al.* (2004 ▶); Gao *et al.* (2005 ▶).
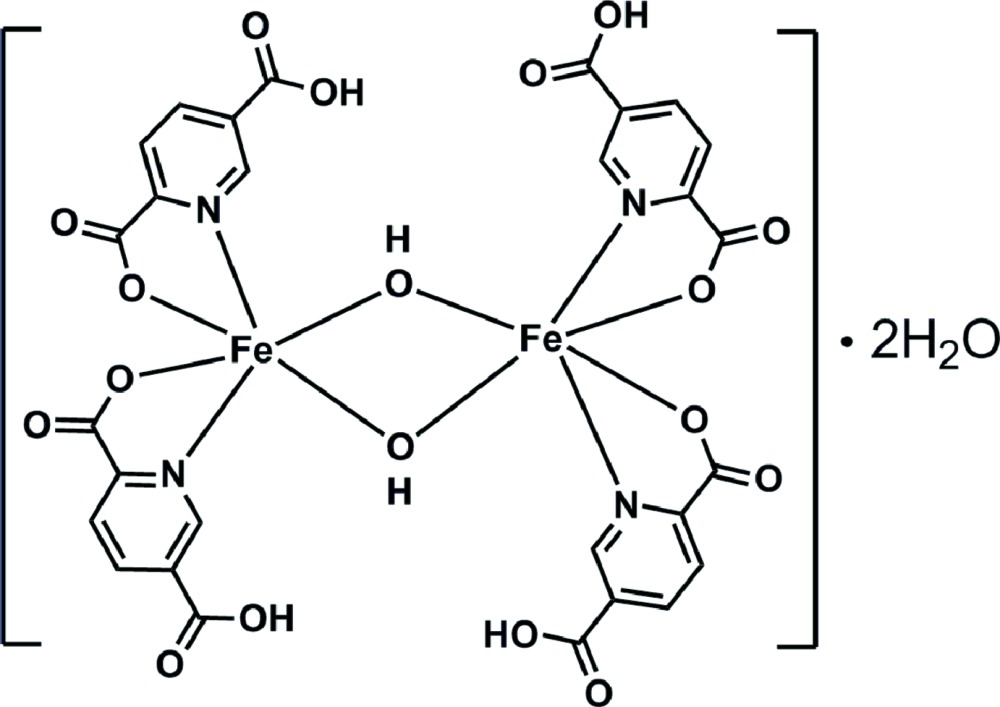


## Experimental

### 

#### Crystal data

[Fe_2_(C_7_H_4_NO_4_)_4_(OH)_2_]·2H_2_O
*M* *_r_* = 846.20
Monoclinic, 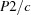

*a* = 7.6130 (7) Å
*b* = 14.2716 (14) Å
*c* = 16.2594 (13) Å
β = 114.556 (4)°
*V* = 1606.8 (3) Å^3^
*Z* = 2
Mo *K*α radiation
μ = 1.00 mm^−1^
*T* = 298 K
0.28 × 0.25 × 0.20 mm

#### Data collection

Bruker APEXII CCD diffractometer
Absorption correction: multi-scan (*SADABS*; Bruker, 2005 ▶) *T*_min_ = 0.767, *T*_max_ = 0.825
11029 measured reflections
3972 independent reflections
3224 reflections with *I* > 2σ(*I*)
*R*_int_ = 0.026

#### Refinement

*R*[*F* ^2^ > 2σ(*F* ^2^)] = 0.035
*wR*(*F* ^2^) = 0.096
*S* = 1.01
3972 reflections
244 parameters
H-atom parameters constrained
Δρ_max_ = 0.37 e Å^−3^
Δρ_min_ = −0.47 e Å^−3^

### 

Data collection: *APEX2* (Bruker, 2005 ▶); cell refinement: *SAINT* (Bruker, 2005 ▶); data reduction: *SAINT*; program(s) used to solve structure: *SHELXS97* (Sheldrick, 2008 ▶); program(s) used to refine structure: *SHELXL97* (Sheldrick, 2008 ▶); molecular graphics: *SHELXTL* (Sheldrick, 2008 ▶); software used to prepare material for publication: *SHELXTL*.

## Supplementary Material

Crystal structure: contains datablock(s) I, New_Global_Publ_Block. DOI: 10.1107/S1600536813029449/rz5089sup1.cif

Structure factors: contains datablock(s) I. DOI: 10.1107/S1600536813029449/rz5089Isup2.hkl

968712

Additional supplementary materials:  crystallographic information; 3D view; checkCIF report

## Figures and Tables

**Table 1:** Hydrogen-bond geometry (Å, °)

*D*—H⋯*A*	*D*—H	H⋯*A*	*D*⋯*A*	*D*—H⋯*A*
O8—H8⋯O2^i^	0.82	1.98	2.761 (2)	160
O4—H4⋯O2^ii^	0.85	1.80	2.643 (2)	175
O9—H9*A*⋯O6^iii^	0.86	1.91	2.735 (2)	162
O10—H10*A*⋯O5	0.87	2.34	2.914 (3)	124
O10—H10*B*⋯O7^iv^	0.88	2.02	2.865 (3)	161
C3—H3⋯O7^v^	0.93	2.36	3.223 (3)	155
C5—H5⋯O10^iii^	0.93	2.54	3.465 (3)	174
C9—H9⋯O8^vi^	0.93	2.53	3.425 (3)	162

Symmetry codes: (i) 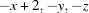; (ii) 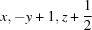; (iii) 
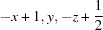
; (iv) 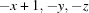; (v) 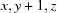; (vi) 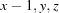.

**Fig. 1.**
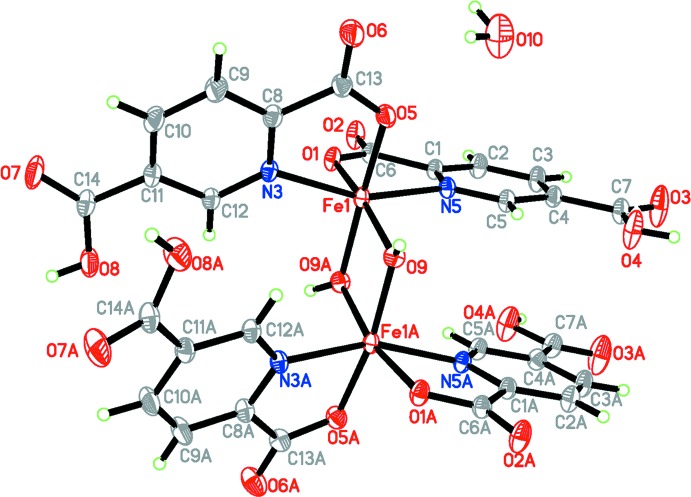
The molecular structure of the title compound showing 30% probability displacement ellipsoids. Symmetry code: (A) -x + 2, y,-z + 1/2.

**Fig. 2.**
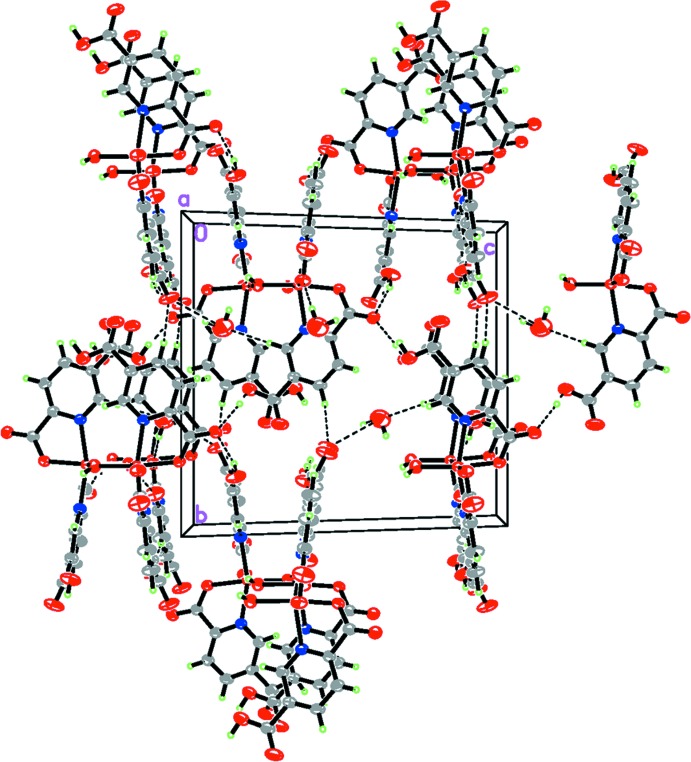
Crystal packing of the title compound viewed down the a axis. Displacement ellipsoids are drawn at the 30% probability level. Hydrogen bonds are shown as dashed lines.

## supplementary crystallographic information

### 1. Comment

In the past few decades, pyridine-2,5-dicarboxylic acid (H_2_pydc) has attracted considerable attention for its ability to coordinate to different metal centres. It can display different kinds of coordination modes, and the relative position of the coordinative moieties is adequate to form supramolecular structures of varied structural features (Zhang *et al.*, 2006; Liang *et al.*, 2000; Wibowo *et al.*, 2011; Zhang *et al.*, 2005). A number of compounds based on pyridine-2,5-dicarboxylic acid and transition metals have been reported, few of them containing Fe ions (Shi *et al.*, 2011; Xu *et al.*, 2004; Gao *et al.*, 2005). Herein, the synthesis and crystal structure of a novel binuclear iron(III) derivative is reported.

As shown in Fig. 1, the metal is coordinated by two O atoms (O1, O5) and two N atoms (N3, N5) from two Hpydc^-^ ligands and two *µ*_2_-OH groups (O9, O9A) to form a slightly distorted octahedral geometry. Two iron metals related by a two-fold axis are bridged by the OH groups to form a binuclear complex molecule. The mean Fe—O and Fe—N distances are 1.971 (9) Å and 2.114 (2) Å, respectively. In the crystal, the title compound features two kinds of hydrogen interactions (O—H···O and C—H···O; Table 1), which connect the binuclear units into a three-dimensional supramolecular network (Fig. 2).

### 2. Experimental

A mixture of pyridine-2,5-dicarboxylic acid (0.0335 g, 0.2 mmol), Sr(OH)_2_·8H_2_O (0.0267 g, 0.1 mmol), Fe(NO_3_)_3_·9H_2_O (0.0404 g, 0.1 mmol), imidazole (0.0235 g, 0.35 mmol), and H_2_O (3 ml, *v*/*v* = 2:1) was sealed in a Pyrex-tube (8 ml) and heated at 120°C for 2 days. The tube was then cooled to room temperature, generating bright-green rod crystals. Yield: 0.0237 g (56%, based on Fe). Elemental analysis calc. for C_28_H_22_Fe_2_N_4_O_20_: C, 39.74; H, 2.62; N, 6.62%. Found: C, 39.66; H, 2.65; N, 6.69%.

### 3. Refinement

Water and hydroxy H atoms were located in a difference Fourier map and refined as riding, with O—H = 0.85-0.88 Å and *U*_iso_(H) = 1.5 *U*_eq_(O). All other H atoms were positioned geometrically and refined as riding with C—H = 0.93 Å and *U*_iso_(H) = 1.2 *U*_eq_ (C).

### Crystal data

**Table:**

[Fe_2_(C_7_H_4_NO_4_)_4_(OH)_2_]·2H_2_O	*F*(000) = 860
*M**_r_* = 846.20	*D*_x_ = 1.749 Mg m^−^^3^
Monoclinic, *P*2/*c*	Mo *K*α radiation, λ = 0.71073 Å
Hall symbol: -P 2yc	Cell parameters from 3376 reflections
*a* = 7.6130 (7) Å	θ = 2.8–27.8°
*b* = 14.2716 (14) Å	µ = 1.00 mm^−^^1^
*c* = 16.2594 (13) Å	*T* = 298 K
β = 114.556 (4)°	Rod, green
*V* = 1606.8 (3) Å^3^	0.28 × 0.25 × 0.20 mm
*Z* = 2	

### Data collection

**Table:**

Bruker APEXII CCD diffractometer	3972 independent reflections
Radiation source: fine-focus sealed tube	3224 reflections with *I* > 2σ(*I*)
Graphite monochromator	*R*_int_ = 0.026
φ and ω scans	θ_max_ = 28.3°, θ_min_ = 2.0°
Absorption correction: multi-scan (*SADABS*; Bruker, 2005)	*h* = −10→9
*T*_min_ = 0.767, *T*_max_ = 0.825	*k* = −19→15
11029 measured reflections	*l* = −21→21

### Refinement

**Table:**

Refinement on *F*^2^	Primary atom site location: structure-invariant direct methods
Least-squares matrix: full	Secondary atom site location: difference Fourier map
*R*[*F*^2^ > 2σ(*F*^2^)] = 0.035	Hydrogen site location: inferred from neighbouring sites
*wR*(*F*^2^) = 0.096	H-atom parameters constrained
*S* = 1.01	*w* = 1/[σ^2^(*F*_o_^2^) + (0.0446*P*)^2^ + 0.9967*P*] where *P* = (*F*_o_^2^ + 2*F*_c_^2^)/3
3972 reflections	(Δ/σ)_max_ < 0.001
244 parameters	Δρ_max_ = 0.37 e Å^−^^3^
0 restraints	Δρ_min_ = −0.47 e Å^−^^3^

### Special details

**Table:**

Geometry. All e.s.d.'s (except the e.s.d. in the dihedral angle between two l.s. planes) are estimated using the full covariance matrix. The cell e.s.d.'s are taken into account individually in the estimation of e.s.d.'s in distances, angles and torsion angles; correlations between e.s.d.'s in cell parameters are only used when they are defined by crystal symmetry. An approximate (isotropic) treatment of cell e.s.d.'s is used for estimating e.s.d.'s involving l.s. planes.
Refinement. Refinement of *F*^2^ against ALL reflections. The weighted *R*-factor *wR* and goodness of fit *S* are based on *F*^2^, conventional *R*-factors *R* are based on *F*, with *F* set to zero for negative *F*^2^. The threshold expression of *F*^2^ > σ(*F*^2^) is used only for calculating *R*-factors(gt) *etc*. and is not relevant to the choice of reflections for refinement. *R*-factors based on *F*^2^ are statistically about twice as large as those based on *F*, and *R*- factors based on ALL data will be even larger.

### Fractional atomic coordinates and isotropic or equivalent isotropic displacement parameters (Å^2^)

**Table:**

	*x*	*y*	*z*	*U*_iso_*/*U*_eq_	
Fe1	0.80977 (4)	0.202541 (19)	0.164770 (19)	0.02479 (10)	
O1	0.7658 (2)	0.21500 (10)	0.03507 (10)	0.0320 (3)	
C1	0.7583 (3)	0.37700 (14)	0.06158 (13)	0.0276 (4)	
O2	0.7390 (3)	0.30988 (11)	−0.07630 (11)	0.0422 (4)	
C2	0.7485 (3)	0.46928 (15)	0.03644 (15)	0.0364 (5)	
H2	0.7459	0.4859	−0.0194	0.044*	
N3	0.7556 (2)	0.05572 (11)	0.14087 (12)	0.0270 (4)	
C3	0.7427 (4)	0.53720 (16)	0.09617 (16)	0.0384 (5)	
H3	0.7356	0.6003	0.0808	0.046*	
O3	0.7248 (4)	0.66606 (13)	0.22363 (14)	0.0732 (7)	
O4	0.7493 (4)	0.55104 (13)	0.31845 (12)	0.0619 (6)	
H4	0.7432	0.5981	0.3492	0.093*	
C4	0.7476 (3)	0.51062 (14)	0.17838 (14)	0.0311 (4)	
O5	0.5359 (2)	0.19337 (10)	0.14846 (11)	0.0346 (3)	
N5	0.7678 (2)	0.35085 (12)	0.14291 (11)	0.0264 (3)	
C5	0.7609 (3)	0.41600 (14)	0.20041 (14)	0.0302 (4)	
H5	0.7651	0.3978	0.2561	0.036*	
O6	0.2935 (2)	0.09752 (13)	0.13321 (15)	0.0517 (5)	
C6	0.7553 (3)	0.29598 (14)	0.00125 (14)	0.0281 (4)	
O7	0.8655 (3)	−0.25246 (13)	0.07024 (15)	0.0579 (5)	
C7	0.7401 (4)	0.58513 (16)	0.24209 (16)	0.0378 (5)	
O8	1.1183 (3)	−0.15856 (12)	0.13517 (14)	0.0515 (5)	
H8	1.1783	−0.2046	0.1309	0.077*	
C8	0.5788 (3)	0.03170 (15)	0.13346 (15)	0.0308 (4)	
O9	0.9102 (2)	0.20283 (10)	0.29588 (10)	0.0295 (3)	
H9A	0.8474	0.1807	0.3246	0.044*	
C9	0.5143 (4)	−0.05918 (16)	0.12100 (19)	0.0442 (6)	
H9	0.3921	−0.0738	0.1172	0.053*	
C10	0.6332 (3)	−0.12806 (16)	0.11428 (18)	0.0435 (6)	
H10	0.5931	−0.1903	0.1065	0.052*	
O10	0.2618 (4)	0.34823 (19)	0.09961 (18)	0.0971 (9)	
H10A	0.2683	0.2904	0.1176	0.146*	
H10B	0.1997	0.3279	0.0440	0.146*	
C11	0.8133 (3)	−0.10417 (15)	0.11911 (15)	0.0322 (4)	
C12	0.8722 (3)	−0.01115 (14)	0.13374 (14)	0.0288 (4)	
H12	0.9945	0.0050	0.1387	0.035*	
C13	0.4555 (3)	0.11238 (16)	0.13878 (15)	0.0333 (5)	
C14	0.9348 (4)	−0.17947 (15)	0.10565 (17)	0.0376 (5)	

### Atomic displacement parameters (Å^2^)

**Table:**

	*U*^11^	*U*^22^	*U*^33^	*U*^12^	*U*^13^	*U*^23^
Fe1	0.03269 (17)	0.01681 (15)	0.02696 (16)	0.00018 (11)	0.01447 (12)	0.00000 (11)
O1	0.0489 (9)	0.0203 (7)	0.0281 (7)	−0.0003 (6)	0.0173 (7)	−0.0018 (6)
C1	0.0343 (10)	0.0215 (9)	0.0284 (10)	0.0000 (8)	0.0144 (8)	−0.0006 (8)
O2	0.0745 (12)	0.0278 (8)	0.0328 (8)	0.0048 (7)	0.0309 (8)	0.0031 (6)
C2	0.0549 (14)	0.0255 (11)	0.0331 (11)	0.0019 (9)	0.0224 (10)	0.0029 (9)
N3	0.0325 (9)	0.0200 (8)	0.0313 (8)	−0.0020 (7)	0.0162 (7)	−0.0021 (7)
C3	0.0579 (14)	0.0200 (10)	0.0414 (12)	0.0024 (9)	0.0247 (11)	0.0012 (9)
O3	0.150 (2)	0.0244 (9)	0.0644 (13)	0.0098 (11)	0.0631 (15)	−0.0020 (9)
O4	0.1225 (18)	0.0296 (10)	0.0442 (10)	0.0052 (10)	0.0452 (12)	−0.0055 (8)
C4	0.0381 (11)	0.0210 (10)	0.0339 (11)	0.0019 (8)	0.0146 (9)	−0.0026 (8)
O5	0.0332 (8)	0.0254 (8)	0.0474 (9)	0.0016 (6)	0.0189 (7)	−0.0015 (6)
N5	0.0342 (9)	0.0195 (8)	0.0274 (8)	0.0024 (7)	0.0149 (7)	0.0011 (6)
C5	0.0399 (11)	0.0241 (10)	0.0285 (10)	0.0023 (8)	0.0163 (9)	−0.0009 (8)
O6	0.0397 (9)	0.0413 (10)	0.0850 (14)	−0.0057 (8)	0.0370 (9)	−0.0127 (10)
C6	0.0339 (10)	0.0232 (10)	0.0295 (10)	0.0004 (8)	0.0156 (8)	−0.0005 (8)
O7	0.0709 (13)	0.0267 (9)	0.0846 (14)	−0.0100 (8)	0.0409 (11)	−0.0218 (9)
C7	0.0531 (14)	0.0255 (11)	0.0384 (12)	0.0043 (9)	0.0226 (11)	−0.0029 (9)
O8	0.0502 (10)	0.0285 (9)	0.0842 (14)	0.0002 (8)	0.0364 (10)	−0.0146 (9)
C8	0.0354 (11)	0.0247 (10)	0.0371 (11)	−0.0025 (8)	0.0197 (9)	−0.0021 (9)
O9	0.0327 (7)	0.0322 (8)	0.0277 (7)	−0.0008 (6)	0.0168 (6)	0.0009 (6)
C9	0.0399 (13)	0.0293 (12)	0.0702 (17)	−0.0096 (10)	0.0295 (12)	−0.0064 (11)
C10	0.0444 (13)	0.0235 (11)	0.0650 (16)	−0.0095 (9)	0.0250 (12)	−0.0078 (11)
O10	0.141 (3)	0.0694 (18)	0.0912 (19)	0.0195 (17)	0.0580 (18)	−0.0053 (15)
C11	0.0399 (11)	0.0216 (10)	0.0360 (11)	−0.0012 (8)	0.0167 (9)	−0.0026 (9)
C12	0.0337 (10)	0.0214 (10)	0.0342 (10)	0.0000 (8)	0.0170 (9)	−0.0007 (8)
C13	0.0341 (11)	0.0315 (11)	0.0383 (11)	0.0001 (9)	0.0190 (9)	−0.0029 (9)
C14	0.0506 (14)	0.0219 (10)	0.0468 (13)	−0.0033 (9)	0.0268 (11)	−0.0038 (9)

### Geometric parameters (Å, º)

**Table:**

Fe1—O9	1.9427 (15)	C4—C7	1.502 (3)
Fe1—O9^i^	1.9543 (15)	O5—C13	1.287 (3)
Fe1—O5	1.9937 (15)	N5—C5	1.335 (3)
Fe1—O1	2.0011 (15)	C5—H5	0.9300
Fe1—N3	2.1397 (17)	O6—C13	1.217 (3)
Fe1—N5	2.1480 (17)	O7—C14	1.201 (3)
O1—C6	1.268 (2)	O8—C14	1.309 (3)
C1—N5	1.347 (3)	O8—H8	0.8200
C1—C2	1.372 (3)	C8—C9	1.372 (3)
C1—C6	1.510 (3)	C8—C13	1.511 (3)
O2—C6	1.231 (2)	O9—Fe1^i^	1.9543 (15)
C2—C3	1.386 (3)	O9—H9A	0.8554
C2—H2	0.9300	C9—C10	1.371 (3)
N3—C12	1.340 (3)	C9—H9	0.9300
N3—C8	1.345 (3)	C10—C11	1.383 (3)
C3—C4	1.375 (3)	C10—H10	0.9300
C3—H3	0.9300	O10—H10A	0.8695
O3—C7	1.187 (3)	O10—H10B	0.8773
O4—C7	1.308 (3)	C11—C12	1.390 (3)
O4—H4	0.8500	C11—C14	1.492 (3)
C4—C5	1.390 (3)	C12—H12	0.9300
			
O9—Fe1—O9^i^	76.25 (7)	N5—C5—C4	121.07 (19)
O9—Fe1—O5	93.39 (6)	N5—C5—H5	119.5
O9^i^—Fe1—O5	169.02 (6)	C4—C5—H5	119.5
O9—Fe1—O1	166.74 (6)	O2—C6—O1	123.53 (19)
O9^i^—Fe1—O1	91.53 (6)	O2—C6—C1	120.67 (18)
O5—Fe1—O1	99.10 (7)	O1—C6—C1	115.78 (18)
O9—Fe1—N3	99.19 (6)	O3—C7—O4	124.3 (2)
O9^i^—Fe1—N3	99.39 (6)	O3—C7—C4	122.8 (2)
O5—Fe1—N3	78.44 (6)	O4—C7—C4	112.89 (19)
O1—Fe1—N3	87.73 (6)	C14—O8—H8	109.5
O9—Fe1—N5	98.23 (6)	N3—C8—C9	122.5 (2)
O9^i^—Fe1—N5	96.84 (6)	N3—C8—C13	115.04 (18)
O5—Fe1—N5	88.13 (6)	C9—C8—C13	122.4 (2)
O1—Fe1—N5	77.87 (6)	Fe1—O9—Fe1^i^	103.75 (7)
N3—Fe1—N5	158.55 (7)	Fe1—O9—H9A	122.9
C6—O1—Fe1	119.39 (13)	Fe1^i^—O9—H9A	127.0
N5—C1—C2	122.20 (19)	C10—C9—C8	118.8 (2)
N5—C1—C6	113.94 (17)	C10—C9—H9	120.6
C2—C1—C6	123.85 (19)	C8—C9—H9	120.6
C1—C2—C3	118.4 (2)	C9—C10—C11	119.4 (2)
C1—C2—H2	120.8	C9—C10—H10	120.3
C3—C2—H2	120.8	C11—C10—H10	120.3
C12—N3—C8	118.98 (17)	H10A—O10—H10B	87.9
C12—N3—Fe1	128.94 (14)	C10—C11—C12	119.1 (2)
C8—N3—Fe1	112.08 (13)	C10—C11—C14	118.3 (2)
C4—C3—C2	119.5 (2)	C12—C11—C14	122.5 (2)
C4—C3—H3	120.3	N3—C12—C11	121.13 (19)
C2—C3—H3	120.3	N3—C12—H12	119.4
C7—O4—H4	105.7	C11—C12—H12	119.4
C3—C4—C5	119.2 (2)	O6—C13—O5	125.6 (2)
C3—C4—C7	118.80 (19)	O6—C13—C8	119.8 (2)
C5—C4—C7	122.0 (2)	O5—C13—C8	114.59 (18)
C13—O5—Fe1	119.57 (13)	O7—C14—O8	124.2 (2)
C5—N5—C1	119.53 (17)	O7—C14—C11	121.3 (2)
C5—N5—Fe1	128.09 (14)	O8—C14—C11	114.47 (19)
C1—N5—Fe1	112.28 (13)		
			
O9—Fe1—O1—C6	−66.9 (3)	C3—C4—C5—N5	−0.4 (3)
O9^i^—Fe1—O1—C6	−89.41 (16)	C7—C4—C5—N5	−179.9 (2)
O5—Fe1—O1—C6	93.34 (16)	Fe1—O1—C6—O2	175.93 (17)
N3—Fe1—O1—C6	171.25 (16)	Fe1—O1—C6—C1	−5.7 (2)
N5—Fe1—O1—C6	7.26 (16)	N5—C1—C6—O2	177.1 (2)
N5—C1—C2—C3	−1.9 (3)	C2—C1—C6—O2	−1.7 (3)
C6—C1—C2—C3	176.8 (2)	N5—C1—C6—O1	−1.3 (3)
O9—Fe1—N3—C12	−91.72 (18)	C2—C1—C6—O1	179.9 (2)
O9^i^—Fe1—N3—C12	−14.28 (18)	C3—C4—C7—O3	2.1 (4)
O5—Fe1—N3—C12	176.66 (19)	C5—C4—C7—O3	−178.5 (3)
O1—Fe1—N3—C12	76.90 (18)	C3—C4—C7—O4	−178.8 (2)
N5—Fe1—N3—C12	124.4 (2)	C5—C4—C7—O4	0.7 (3)
O9—Fe1—N3—C8	87.60 (15)	C12—N3—C8—C9	1.6 (3)
O9^i^—Fe1—N3—C8	165.05 (14)	Fe1—N3—C8—C9	−177.8 (2)
O5—Fe1—N3—C8	−4.01 (14)	C12—N3—C8—C13	−177.72 (18)
O1—Fe1—N3—C8	−103.78 (15)	Fe1—N3—C8—C13	2.9 (2)
N5—Fe1—N3—C8	−56.3 (2)	O9^i^—Fe1—O9—Fe1^i^	−0.25 (9)
C1—C2—C3—C4	0.2 (4)	O5—Fe1—O9—Fe1^i^	176.08 (6)
C2—C3—C4—C5	0.9 (4)	O1—Fe1—O9—Fe1^i^	−23.5 (3)
C2—C3—C4—C7	−179.6 (2)	N3—Fe1—O9—Fe1^i^	97.25 (7)
O9—Fe1—O5—C13	−93.78 (17)	N5—Fe1—O9—Fe1^i^	−95.32 (7)
O9^i^—Fe1—O5—C13	−74.7 (4)	N3—C8—C9—C10	−1.1 (4)
O1—Fe1—O5—C13	90.68 (17)	C13—C8—C9—C10	178.1 (2)
N3—Fe1—O5—C13	4.91 (16)	C8—C9—C10—C11	−0.8 (4)
N5—Fe1—O5—C13	168.08 (17)	C9—C10—C11—C12	2.2 (4)
C2—C1—N5—C5	2.4 (3)	C9—C10—C11—C14	−176.1 (2)
C6—C1—N5—C5	−176.44 (18)	C8—N3—C12—C11	−0.1 (3)
C2—C1—N5—Fe1	−174.31 (18)	Fe1—N3—C12—C11	179.16 (15)
C6—C1—N5—Fe1	6.9 (2)	C10—C11—C12—N3	−1.7 (3)
O9—Fe1—N5—C5	−16.69 (18)	C14—C11—C12—N3	176.5 (2)
O9^i^—Fe1—N5—C5	−93.71 (18)	Fe1—O5—C13—O6	175.8 (2)
O5—Fe1—N5—C5	76.46 (18)	Fe1—O5—C13—C8	−4.7 (3)
O1—Fe1—N5—C5	176.19 (19)	N3—C8—C13—O6	−179.6 (2)
N3—Fe1—N5—C5	127.3 (2)	C9—C8—C13—O6	1.1 (4)
O9—Fe1—N5—C1	159.65 (14)	N3—C8—C13—O5	0.9 (3)
O9^i^—Fe1—N5—C1	82.63 (14)	C9—C8—C13—O5	−178.4 (2)
O5—Fe1—N5—C1	−107.19 (14)	C10—C11—C14—O7	18.1 (4)
O1—Fe1—N5—C1	−7.47 (14)	C12—C11—C14—O7	−160.1 (2)
N3—Fe1—N5—C1	−56.3 (2)	C10—C11—C14—O8	−162.0 (2)
C1—N5—C5—C4	−1.2 (3)	C12—C11—C14—O8	19.8 (3)
Fe1—N5—C5—C4	174.93 (15)		

Symmetry code: (i) −*x*+2, *y*, −*z*+1/2.

### Hydrogen-bond geometry (Å, º)

**Table:**

*D*—H···*A*	*D*—H	H···*A*	*D*···*A*	*D*—H···*A*
O8—H8···O2^ii^	0.82	1.98	2.761 (2)	160
O4—H4···O2^iii^	0.85	1.80	2.643 (2)	175
O9—H9*A*···O6^iv^	0.86	1.91	2.735 (2)	162
O10—H10*A*···O5	0.87	2.34	2.914 (3)	124
O10—H10*B*···O7^v^	0.88	2.02	2.865 (3)	161
C3—H3···O7^vi^	0.93	2.36	3.223 (3)	155
C5—H5···O10^iv^	0.93	2.54	3.465 (3)	174
C9—H9···O8^vii^	0.93	2.53	3.425 (3)	162

Symmetry codes: (ii) −*x*+2, −*y*, −*z*; (iii) *x*, −*y*+1, *z*+1/2; (iv) −*x*+1, *y*, −*z*+1/2; (v) −*x*+1, −*y*, −*z*; (vi) *x*, *y*+1, *z*; (vii) *x*−1, *y*, *z*.
